# Early prognostication for ICU patients with combined respiratory and circulatory failure: an interpretable machine learning approach

**DOI:** 10.1038/s41598-026-59064-y

**Published:** 2026-06-25

**Authors:** Adam Piasecki, Kamil Adamczyk, Marcin Śniegowski, Raffaele Mandarano, Beatrice Brunoni, Harm-Jan de Grooth, Paul W. G. Elbers

**Affiliations:** 1 Department of Anesthesiology and Intensive Care, Institute of Clinical Sciences at the Sahlgrenska Academy, University of Gothenburg, Gothenburg, Sweden; 2 Department of Anesthesiology and Intensive Care, National Medical Institute of the Ministry of the Interior and Administration, Warsaw, Poland; 3 Department of Computer Science, National Medical Institute of the Ministry of the Interior and Administration, Warsaw, Poland; 4 Department of Anesthesiology and Intensive Care, Careggi University Hospital, Florence, Italy; 5 Department of Medicine and Surgery, University of Milan-Bicocca, Monza, Monza-Brianza Italy; 6 Intensive Care Center, UMC Utrecht, Utrecht, The Netherlands; 7 Department of Intensive Care Medicine, Amsterdam Medical Data Science, Amsterdam Public Health, Amsterdam UMC, Vrije Universiteit, Amsterdam, The Netherlands

**Keywords:** Computational biology and bioinformatics, Diseases, Health care, Mathematics and computing, Medical research, Risk factors

## Abstract

Accurate prognostication in the intensive care unit (ICU) is essential for delivering personalized and ethically sound care, yet it remains a challenge for high-risk patients. This study aimed to develop and validate an exploratory machine learning model for predicting ICU mortality in critically ill patients requiring both mechanical ventilation and vasoactive support. We conducted a retrospective analysis of 4816 patient records from the AmsterdamUMCdb database. The XGBoost model was developed using 66 clinical and laboratory features collected within the first 24 h of ICU admission. The model demonstrated robust discriminatory performance in predicting in-ICU mortality at 24h from admission, achieving in internal validation an area under the receiver operating characteristic curve (AUROC) of 0.831 (95% CI [0.799–0.860]), a macro F1 score of 0.735, and a Brier score of 0.130, indicating reliable calibration. Explainable AI methods identified the minimum Glasgow Coma Scale score, patient age, and minimum platelet count as the most significant predictors. In this exploratory study, a machine learning model using single-center early ICU data showed promising performance for mortality prediction in a high-risk population. Given the lack of external or prospective validation, the model should be considered investigational and requires further validation before clinical application.

**Supplementary Information:**

The online version contains supplementary material available at 10.1038/s41598-026-59064-y.

## Introduction

Prognostication in the intensive care unit (ICU) is crucial for delivering personalized, efficient and ethically sound care^1^. While prognostication can address a range of outcomes, the high burden of critical illness and a global ICU mortality rate of approximately 16%^2^ position mortality prediction as a primary objective.

To aid in the early identification of high-risk patients and compare mortality between ICUs several scoring systems have been developed. Tools such as the Acute Physiology and Chronic Health Evaluation (APACHE III)^3^, Simplified Acute Physiology Score (SAPS 3)^4,5^, and Mortality Probability Model (MPM III)^6^ estimate outcomes, including ICU mortality, based on illness severity at admission^7^. However, the complexity and dynamic nature of critical illness, combined with the high-stakes, time-sensitive ICU environment and limitations of existing scoring systems, often make accurate prognostication challenging. Also, physicians’ capacity to process large volumes of clinical data is limited, and human-based judgement is often unreliable^8,9^.

In recent years, advances in computational power and data availability, enabled the use of Artificial Intelligence (AI) for data-driven predictive modeling. Machine learning (ML), a subset of AI, offers advantages over conventional methods through its ability to process high-dimensional, complex data obtained from electronic health records (EHRs) and model non-linear relationships. Thus, ML models show promise as supportive tools in personalized care, facilitating shared decision-making and potentially improving patient outcomes^10–12^. Machine learning applications have been widely described in healthcare^13^, including in the ICU across a range of domains^11,14^.

Compared to conventional illness severity scores, ML has shown superior performance in predicting ICU mortality across various cohorts, including patients with severe pneumonia^15^, sepsis^16^, and all-cause admissions^17^. This is further supported by a recent meta-analysis demonstrating that ML models offer enhanced predictive accuracy over traditional severity scoring systems^18^.

Patients requiring both mechanical ventilation and vasoactive therapy are most frequent in the ICU and represent a distinct phenotype of critical illness associated with high mortality^19^; higher mortality is observed only when CRRT is also used^19^. The extent of organ support significantly influences outcomes: patients receiving vasopressors and/or mechanical ventilation have higher illness severity scores and in-hospital mortality compared to those not receiving such interventions^20^. Furthermore, patients with both respiratory and cardiovascular failure have a fourfold higher risk of ICU death than those with isolated respiratory failure^21^. Despite the clinical significance of this subgroup, dedicated prognostic ML models are lacking. Existing tools generally target the broader ICU population^22^, ventilated patients^23–26^ or specific conditions^15,16^, failing to capture the unique risk profile of patients requiring respiratory and cardiovascular organ support. Addressing this gap, focusing exclusively on the mechanically ventilated and vasopressor/inotrope drug-treated population, is essential to better stratify risk, tailor interventions, allocate ICU resources efficiently, and guide shared decision-making processes.

Predictive models in this context are intended to support, not replace, clinical judgment, and any identified risk factors should be interpreted within the clinical context rather than as direct therapeutic targets.

This exploratory study developed and internally evaluated an interpretable machine learning model to predict in-ICU mortality at 24 h from admission in patients receiving both mechanical ventilation and vasopressor or inotropic therapy.

## Results

### Dataset

The mean (SD) age of the overall cohort was 63.1 (15.6) years. Non-survivors were older than survivors, with a mean age of 66.9 (14.5) years compared with 61.8 (15.8) years, respectively (Standardized Mean Difference, SMD = 0.337).

Gender distribution was similar between groups, with males representing the majority in both survivors (63.4%) and non-survivors (62.8%) (SMD = 0.017).

Differences were observed in admission reasons. Non-survivors were more frequently admitted acutely (10.9% vs. 5.0%) and for medical reasons (27.3% vs. 18.8%) compared with survivors. In contrast, elective surgery admissions were more common among survivors (8.5% vs. 1.7%), and emergency surgery was also slightly more frequent among survivors (12.2% vs. 9.5%) (SMD = 0.430).

Regarding specialty categories, non-survivors were more often admitted via cardiology (23.6% vs. 13.0%) and other medical specialties (27.5% vs. 20.7%). Conversely, survivors were more frequently admitted through cardiac surgery (19.3% vs. 7.9%) and other surgical specialties (38.1% vs. 28.5%) (SMD = 0.475).

The mean (SD) time from ICU admission to clinical deterioration was similar between groups, on average 1.59 (3.37) hours in the overall cohort.

In survivors, the median length of ICU stay was 216 h (Interquartile Range, IQR [120–432]), for non-survivors the median time to death was 192 h (IQR [72–384]).

Overall 3581 patients (74.36%) were discharged from the ICU alive, while 1235 (25.64%) did not survive to ICU discharge.

Baseline patient characteristics and predictors, aggregated over the 24-h period following ICU admission, and stratified by survival status are presented in Table 1.

**Table 1:** Patient characteristics between survivors and non-survivors in the study cohort from the AmsterdamUMCdb database. The data represents predictors used for model development.

	Overall	Survivor	Non-survivor	SMD
n	4816	3581	1235	
Age (years) (mean (SD))	63.11 (15.63)	61.80 (15.80)	66.91 (14.48)	0.337
Gender (%)				0.017
Female	1668 ( 34.6)	1233 ( 34.4)	435 ( 35.2)	
Male	3047 ( 63.3)	2272 ( 63.4)	775 ( 62.8)	
Missing	101 ( 2.1)	76 ( 2.1)	25 ( 2.0)	
Admission_reason (%)				0.430
Missing	2549 ( 52.9)	1935 ( 54.0)	614 ( 49.7)	
Acute admission	314 ( 6.5)	180 ( 5.0)	134 ( 10.9)	
Elective admission	66 ( 1.4)	54 ( 1.5)	12 ( 1.0)	
Elective surgery	324 ( 6.7)	303 ( 8.5)	21 ( 1.7)	
Emergency surgery	554 ( 11.5)	437 ( 12.2)	117 ( 9.5)	
Medical reasons	1009 ( 21.0)	672 ( 18.8)	337 ( 27.3)	
Specialty_category (%)				0.475
Cardiac surgery	788 ( 16.4)	690 ( 19.3)	98 ( 7.9)	
Cardiology	757 ( 15.7)	466 ( 13.0)	291 ( 23.6)	
Critical care	476 ( 9.9)	322 ( 9.0)	154 ( 12.5)	
Other medical	1080 ( 22.4)	740 ( 20.7)	340 ( 27.5)	
Other surgery	1715 ( 35.6)	1363 ( 38.1)	352 ( 28.5)	
HR_twa_over (mean (SD))	1.99 (3.98)	1.90 (3.89)	2.25 (4.24)	0.088
SBP_twa_under (mean (SD))	0.49 (1.11)	0.46 (1.01)	0.58 (1.35)	0.104
DBP_twa_under (mean (SD))	3.07 (3.04)	3.02 (2.97)	3.20 (3.21)	0.059
MAP_twa_under (mean (SD))	0.43 (0.78)	0.42 (0.75)	0.48 (0.87)	0.085
SpO2_min (%) (mean (SD))	94.13 (1.90)	94.18 (1.91)	93.99 (1.86)	0.097
Temperature_max (℃) (mean (SD))	36.83 (1.29)	36.96 (1.16)	36.46 (1.57)	0.360
FiO2_max (%) (mean (SD))	60.02 (14.09)	59.30 (13.96)	62.15 (14.26)	0.203
PEEP_max (cmH_2_0) (mean (SD))	10.27 (4.08)	10.06 (4.06)	10.90 (4.07)	0.207
PPeak_max (cmH_2_0) (mean (SD))	27.80 (7.68)	27.28 (7.65)	29.33 (7.60)	0.269
MV_mean (L/min) (mean (SD))	8.67 (1.85)	8.62 (1.83)	8.83 (1.92)	0.114
PaO2_FiO2_ratio_min (mean (SD))	173.89 (95.92)	176.42 (98.38)	166.25 (87.72)	0.109
Ventilation_mode_category (%)				0.103
Missing	13 ( 0.3)	7 ( 0.2)	6 ( 0.5)	
Assisted Ventilation	85 ( 1.8)	64 ( 1.8)	21 ( 1.7)	
Controlled Ventilation	1819 ( 37.8)	1319 ( 36.8)	500 ( 40.5)	
MMV	7 ( 0.1)	5 ( 0.1)	2 ( 0.2)	
Other/Special Modes	53 ( 1.1)	37 ( 1.0)	16 ( 1.3)	
Pressure Support/CPAP	2641 ( 54.8)	2005 ( 56.0)	636 ( 51.5)	
SIMV	198 ( 4.1)	144 ( 4.0)	54 ( 4.4)	
CVP_min (mmHg) (mean (SD))	6.43 (3.44)	6.22 (3.42)	7.03 (3.44)	0.237
Urine_output_min (ml/h) (mean (SD))	23.36 (25.38)	25.04 (25.43)	18.45 (24.60)	0.263
GCS_min (mean (SD))	12.75 (2.49)	13.19 (2.10)	11.41 (3.05)	0.679
RASS_scale_min (mean (SD))	− 2.77 (1.95)	− 2.54 (1.95)	− 3.56 (1.73)	0.557
RASS_scale_max (mean (SD))	− 2.77 (1.95)	− 2.54 (1.95)	− 3.56 (1.73)	0.557
Ramsay_scale_min (mean (SD))	5.97 (0.08)	5.96 (0.08)	5.98 (0.06)	0.297
Ramsay_scale_max (mean (SD))	5.97 (0.08)	5.96 (0.08)	5.98 (0.06)	0.297
pH_min (mean (SD))	7.27 (0.09)	7.28 (0.08)	7.26 (0.10)	0.225
pO2_min (mmHg) (mean (SD))	62.32 (25.99)	62.92 (26.59)	60.58 (24.09)	0.092
pCO2_max (mmHg) (mean (SD))	48.71 (7.47)	48.65 (7.31)	48.87 (7.92)	0.029
HCO3_min (mean (SD))	19.55 (3.80)	19.82 (3.67)	18.75 (4.04)	0.278
Lactate_max (mmol/L) (mean (SD))	2.90 (1.75)	2.73 (1.68)	3.35 (1.86)	0.347
Glucose_mean (mmol/L) (mean (SD))	8.12 (1.47)	8.10 (1.44)	8.17 (1.55)	0.052
Sodium_mean (mmol/L) (mean (SD))	139.68 (4.22)	139.63 (4.13)	139.82 (4.47)	0.046
Potassium_mean (mmol/L) (mean (SD))	4.11 (0.37)	4.11 (0.37)	4.11 (0.38)	0.002
Phosphate_min (mmol/L) (mean (SD))	0.95 (0.37)	0.94 (0.36)	0.98 (0.39)	0.100
Chloride_min (mmol/L) (mean (SD))	106.33 (5.73)	106.51 (5.68)	105.85 (5.82)	0.116
Creatinine_max (μmol/L) (mean (SD))	111.35 (49.38)	106.42 (46.97)	125.59 (53.28)	0.382
Hemoglobin_min (mmol/L) (mean (SD))	6.08 (1.25)	6.08 (1.23)	6.09 (1.31)	0.006
LEU_max (× 10^9^/L) (mean (SD))	14.74 (6.31)	14.75 (6.12)	14.70 (6.84)	0.007
LYMPH_max (× 10^9^/L) (mean (SD))	1.00 (0.58)	1.04 (0.59)	0.89 (0.53)	0.251
MONOCYT_max (× 10^9^/L) (mean (SD))	0.68 (0.44)	0.71 (0.43)	0.62 (0.44)	0.196
PLT_min (× 10^9^/L) (mean (SD))	164.45 (82.79)	165.30 (79.97)	161.96 (90.47)	0.039
INR_max (mean (SD))	1.54 (0.35)	1.52 (0.34)	1.60 (0.37)	0.241
APTT_max (sec) (mean (SD))	52.51 (16.04)	51.02 (15.63)	56.78 (16.42)	0.359
Bilirubin_total_max (μmol/L) (mean (SD))	12.16 (6.66)	11.99 (6.65)	12.60 (6.66)	0.092
ALAT_max (U/L) (mean (SD))	71.57 (68.76)	67.43 (66.25)	82.19 (73.79)	0.211
ASPAT_max (U/L) (mean (SD))	119.24 (123.25)	108.69 (116.05)	146.34 (136.45)	0.297
Albumin_min (g/L) (mean (SD))	16.09 (10.02)	16.24 (10.11)	15.69 (9.76)	0.055
Troponin_max (μg/L) (mean (SD))	0.60 (0.75)	0.58 (0.74)	0.64 (0.76)	0.088
Creatine_kinase_max (U/L) (mean (SD))	38.71 (37.85)	37.00 (36.79)	43.59 (40.37)	0.171
CRP_max (mg/L) (mean (SD))	100.70 (102.08)	99.81 (102.80)	103.12 (100.13)	0.033
Metronidazole = Yes (%)	601 ( 25.9)	459 ( 27.2)	142 ( 22.3)	0.114
Ceftriaxone = Yes (%)	1478 ( 63.7)	1076 ( 63.8)	402 ( 63.2)	0.013
Colistin = No (%)	2322 (100.0)	1686 (100.0)	636 (100.0)	< 0.001
Meropenem = Yes (%)	77 ( 3.3)	44 ( 2.6)	33 ( 5.2)	0.134
Imipenem = Yes (%)	175 ( 7.5)	98 ( 5.8)	77 ( 12.1)	0.222
Vancomycin = Yes (%)	492 ( 21.2)	381 ( 22.6)	111 ( 17.5)	0.129
Gentamicin = Yes (%)	206 ( 8.9)	158 ( 9.4)	48 ( 7.5)	0.066
Amikacin = Yes (%)	1 ( 0.0)	1 ( 0.1)	0 ( 0.0)	0.034
Ciprofloxacin = Yes (%)	266 ( 11.5)	171 ( 10.1)	95 ( 14.9)	0.145
Linezolid = Yes (%)	1 ( 0.0)	0 ( 0.0)	1 ( 0.2)	0.056
Fluconazole = Yes (%)	178 ( 7.7)	122 ( 7.2)	56 ( 8.8)	0.058
Anidulafungin = Yes (%)	47 ( 2.0)	26 ( 1.5)	21 ( 3.3)	0.115
Voriconazole = Yes (%)	91 ( 3.9)	55 ( 3.3)	36 ( 5.7)	0.116
Noradrenaline_mg_per_hour_max (mean (SD))	1.10 (1.05)	1.02 (1.02)	1.33 (1.11)	0.287
Hydrocortisone = No (%)	2322 (100.0)	1686 (100.0)	636 (100.0)	< 0.001
Albumin_iv = Yes (%)	164 ( 7.1)	107 ( 6.3)	57 ( 9.0)	0.099
Transfusion = Yes (%)	1318 ( 27.5)	943 ( 26.5)	375 ( 30.5)	0.090
Time_from_admission_to_deterioration_mean (h) (mean (SD))	1.59 (3.37)	1.59 (3.35)	1.59 (3.43)	< 0.001

HR, Heart Rate; SBP, Systolic Blood Pressure; DBP, Diastolic Blood Pressure; MAP, Mean Arterial Pressure; FiO2, Fraction of Inspired Oxygen; PEEP, Positive End-Expiratory Pressure; PPeak, Peak Inspiratory (Airway) Pressure; MV, Minute Ventilation; PaO2_FiO2_ratio, Arterial Oxygen Partial Pressure to Fraction of Inspired Oxygen Ratio (P/F ratio); MMV, Mandatory Minute Ventilation; CPAP, Continuous Positive Airway Pressure; SIMV, Synchronized Intermittent Mandatory Ventilation; CO, Cardiac Output; CVP, Central Venous Pressure; PAP, Pulmonary Artery Pressure; PCWP, Pulmonary Capillary Wedge Pressure; SVV, Stroke Volume Variation; GCS, Glasgow Coma Scale; RASS, Richmond Agitation–Sedation Scale; SD, standard deviation; SMD, standardized mean difference; values of 0.1, 0.2, and 0.5 indicate small, moderate, and large differences between groups.

### Hyperparameters tuning

Running 1000 trials with Optuna to identify the optimal set of model hyperparameters within the defined search space resulted in an AUROC of 0.830.

The optimized XGBoost hyperparameters were: max_depth = 6, learning_rate = 0.210, subsample = 0.983, colsample_bytree = 0.886, colsample_bylevel = 0.814, colsample_bynode = 0.978, min_child_weight = 19, λ = 0.000, α = 0.107, gamma = 1.340, and n_estimators = 213.

The optimization history, hyperparameters, and their values are presented in Supplementary Material Figure S1.

### Discrimination

**Fig. 1:**
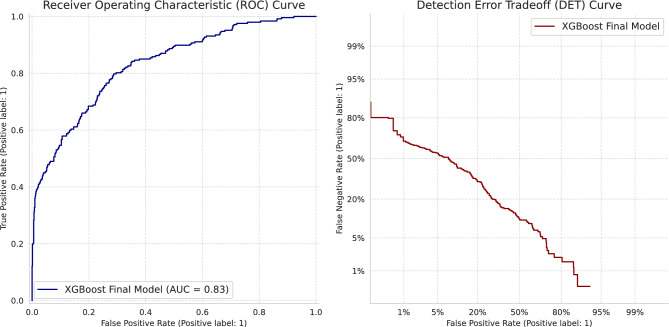
The receiver operating characteristic curve (left panel) and the detection error tradeoff curve (right panel) for the final XGBoost model. The model’s outcome was ICU mortality, a binary variable where 0 represents survival to ICU discharge and 1 (Positive label) indicates death within the ICU. AUC, Area Under Curve.

The final model exhibited robust discriminatory performance on the test set, achieving an AUROC of 0.831 (95% CI [0.799–0.860]) (Fig. 1a), a positive predictive value (PPV) of 0.721 (95% CI [0.647–0.788]) and sensitivity of 0.492 (95% CI [0.431–0.552]). Specificity was at 0.934 (95% CI [0.916–0.951]), while the negative predictive value (NPV) was 0.842 (95% CI [0.818–0.865]). Classification metrics were computed using the default decision threshold of 0.5. The detection error curve further illustrates the trade-off between false positive and false negative rates across different decision thresholds (Fig. 1b). An appropriate balance between PPV and sensitivity was maintained across all classes, with a macro F1 score of 0.735 (95% CI [0.701–0.764]).

### Calibration

**Fig. 2:**
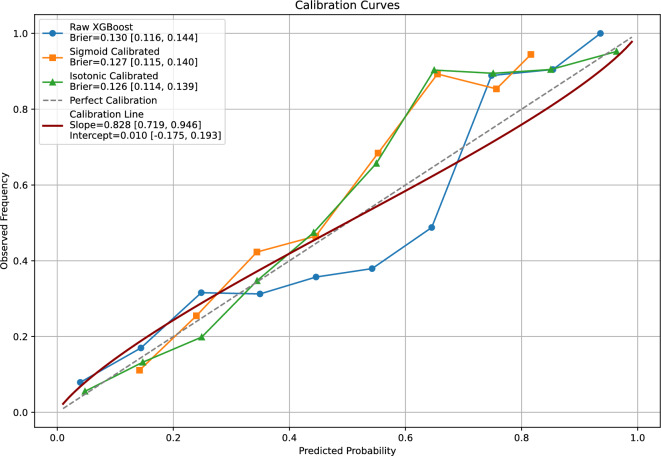
Calibration curves for the final XGBoost model before and after applying post-hoc probability calibration using sigmoid and isotonic methods. Brier, Brier score. XGBoost, Extreme Gradient Boosting algorithm.

Figure 2 presents the calibration curves for the final XGBoost model before and after applying post-hoc probability calibration using sigmoid and isotonic methods.

The raw (uncalibrated) model demonstrated a calibration slope of 0.828 (95% CI [0.719–0.946]) and calibration-in-the-large of 0.010 (95% CI [-0.175 to 0.193]). Sigmoid calibration resulted in a slope of 1.452 (95% CI [1.282–1.628]) and intercept of 0.522 (95% CI [0.236–0.802]), while isotonic calibration yielded a slope of 1.215 (95% CI [1.037–1.416]) and intercept of 0.265 (95% CI [0.009–0.511]). Although calibrated models demonstrated slightly lower Brier scores (isotonic 0.126 (95% CI [0.114–0.139]) and sigmoid 0.127 (95% CI [0.115–0.140]) vs. raw 0.130 (95% CI [0.116–0.144])), the raw model exhibited calibration parameters closest to the ideal values (slope = 1, intercept = 0) and was therefore regarded as optimal. The very similar Brier scores indicate comparable overall performance, while the slight deterioration in calibration metrics after post-hoc calibration may reflect mild overfitting during the calibration process.

### Model interpretability

**Fig. 3:**
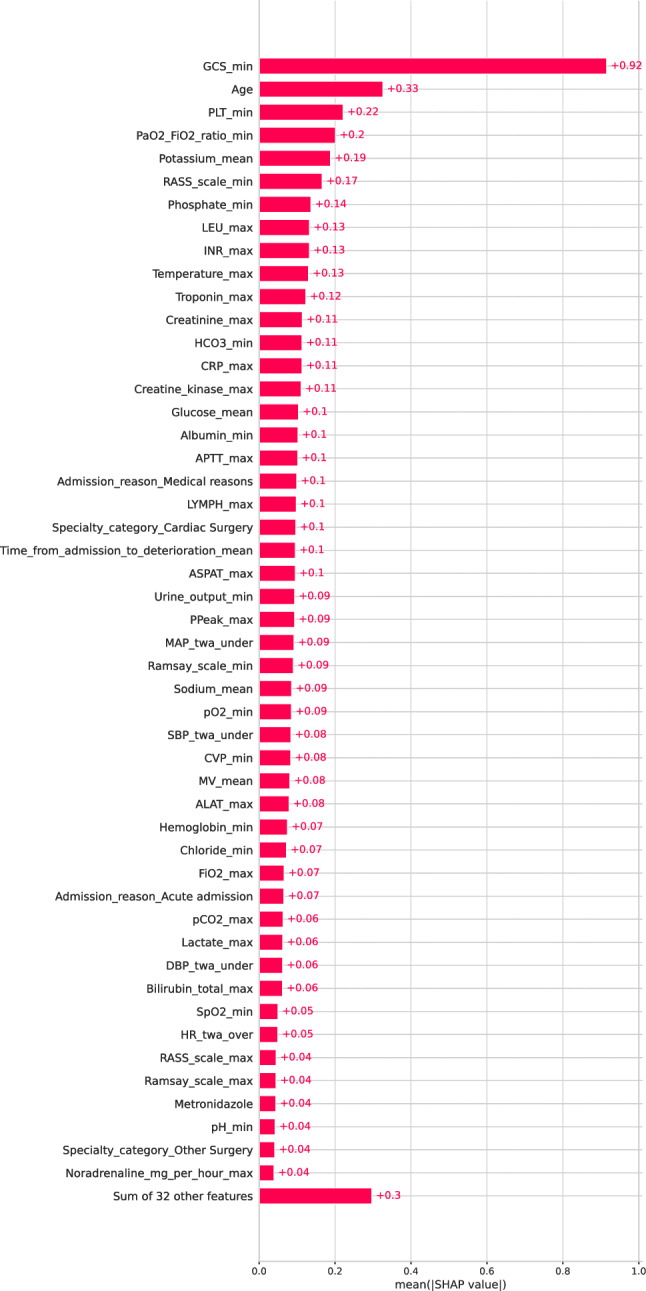
Global feature importance for ICU mortality using SHapley Additive exPlanations (SHAP). Bars show each feature’s contribution to the model output computed on the test cohort, based on the mean absolute SHAP value.

**Fig. 4:**
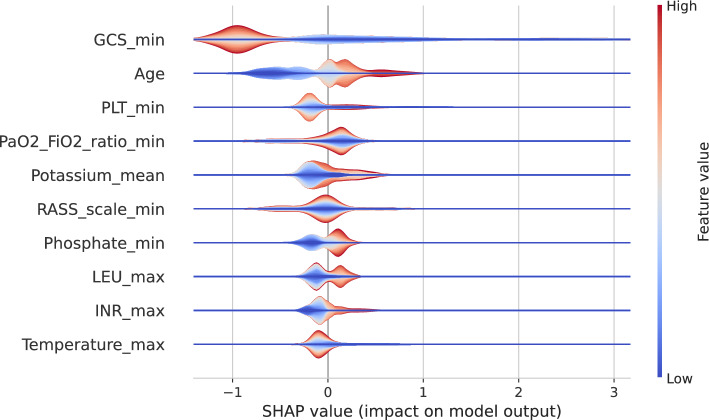
SHapley Additive exPlanations (SHAP) violin plot of feature effects on ICU mortality. For each feature, the distribution of SHAP values shows its effect on the model output: positive values increase predicted mortality, negative values decrease it. Computed on the test cohort.

**Fig. 5:**
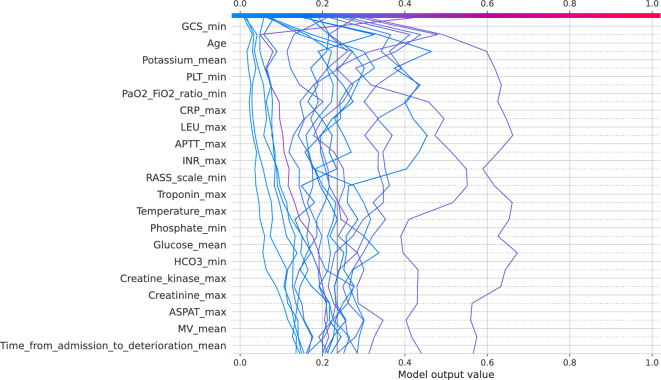
SHapley Additive exPlanations (SHAP) decision tree plot depicting cumulative feature effects on ICU mortality. Each line represents one randomly selected patient in the test set; to aid readability, we display 10 patients per outcome class (survivor/non-survivor). Lines are starting at the model’s baseline and moving across features ordered by global importance.

The SHAP analysis identified the most influential parameters, with the minimum score of GCS, age and minimum platelet count emerging as key contributors to the model’s decision-making process. Figures 3 and 4 illustrate the global feature importance, ranked in descending order, as shown by the SHAP bar plot and beeswarm plot, respectively. Figure 5 presents the SHAP decision plot for 10 randomly selected instances from each outcome group (survivors and non-survivors). The feature importance order depicted in this figure reflects individual contributions for these instances and does not correspond to the global ranking shown in the beeswarm or bar plots.

### Internal validation

The final model demonstrated robust performance on both the training and cross-validation datasets, as indicated by relatively high macro F1 scores (Supplementary Material Figure S2). This suggests low bias, and the relatively small difference between the training and cross-validation macro F1 scores indicates minimal variance. Thus, the model does not seem to be overfitting.

## Discussion and conclusions

### Statement of major findings

This study developed and evaluated a machine learning (ML) model for predicting in-ICU mortality at 24 h from ICU admission in patients requiring both mechanical ventilation and vasoactive therapy. The model, based on the XGBoost algorithm with hyperparameters optimized via the Optuna framework, was trained on 2889, validated on 963, and tested on 964 patients from the AmsterdamUMC database. The final model achieved an AUROC of 0.831 (95% CI [0.799–0.860]), sensitivity of 0.492 (95% CI [0.431–0.552]), specificity of 0.934 (95% CI [0.916–0.951]), and a macro F1 score of 0.735 (95% CI [0.701–0.764]). SHAP analysis identified minimum Glasgow Coma Scale (GCS) score, age, and minimum platelet count as the most influential predictors.

### Comparison with previous studies

The application of ML for early mortality prediction in ICU settings has expanded rapidly in recent years, leading to the development of various models targeting different patient populations. Our model, specifically designed for patients receiving both mechanical ventilation and vasopressor/inotrope support, achieved an AUROC of 0.831.

Iwase et al.^22^ reported a predictive algorithm for ICU mortality and length of stay with a notably high AUROC of 0.945 using Random Forest. However, their model was trained using data from the first 24 h post-ICU admission in all patients, whereas our study targeted a more critically ill subgroup—patients requiring both ventilatory and vasoactive support with data collected during the first 24 h from admission.

Furthermore, in the study by Iwase et al., the top predictors varied across outcomes (mortality, short- and long-term length of stay), and the predictors for mortality (lactate, LDH, and PLT count) differ from those identified in our study (minimum GCS, age, and PLT).

The Glasgow Coma Scale (GCS) has been consistently validated as a strong and independent predictor of in-hospital and ICU mortality in critically ill patients, and corroborated by multiple studies demonstrating that lower GCS scores are significantly associated with increased risk of death across diverse ICU populations^27,28^.

Advanced age has been consistently demonstrated to correlate with elevated in-hospital and ICU mortality rates among patients admitted to intensive care units. Indeed, age is a ubiquitous variable in established prognostic models such as APACHE II and SAPS II^29^, as it serves as a proxy for diminished physiological reserve, a greater burden of comorbidities, and a compromised capacity for recovery^30^.

Thrombocytopenia on ICU admission or during early ICU stay is a strong predictor of mortality, as it reflects underlying critical illness severity, systemic inflammation, sepsis, or hematologic dysfunction. Multiple studies have shown that low platelet counts are independently associated with higher in-hospital and ICU mortality rates, particularly in patients with sepsis, trauma, or multi-organ failure^31,32^, making it a key biomarker in critical care prognostication.

The combined contribution of GCS, age and PLT, identified in our study within the specific high-risk subgroup of patients requiring combined respiratory-vasoactive support, likely reflects the interaction between acute neurologic dysfunction, underlying patient vulnerability and systemic derangements such as coagulopathy and/or inflammation. This constellation may highlight aspects that models derived from more heterogeneous ICU populations may not fully capture. Also, in line with personalized medicine, a one-size-fits-all modeling approach may be insufficient and targeting specific outcomes or patient populations may offer a meaningful advantage.

Of note, SHAP-based feature importance demonstrated in this study reflect non-causal, predictive associations and are intended to enhance model transparency rather than establish mechanistic relationships. While age is non-modifiable, it remains prognostically informative as a marker of diminished physiological reserve. Minimum GCS and platelet count, although not directly treatable, reflect underlying processes such as neurological dysfunction and coagulopathy that may guide targeted interventions. These findings may serve as a basis for hypothesis generation in future studies employing causal frameworks.

Although our overall AUROC was lower than that reported by Iwase et al., the macro F1 score of 0.735 indicates reliable performance and balanced classification in the high-risk population of patients on mechanical ventilation and vasopressors.

Similarly, Li et al. developed ML-based models to predict mortality in ICU patients with heart failure using the MIMIC-III database, reporting favorable discrimination and calibration with XGBoost and LASSO regression^33^. Our model’s AUROC of 0.831 and macro F1 score of 0.735 are consistent with their findings.

In another relevant study, Suttapanit et al.^34^ reported an AUROC of 0.77 for the National Early Warning Score (NEWS) in predicting 28-day mortality in sepsis patients. In contrast, our model achieved a substantially higher AUROC of 0.831 in a population requiring both ventilatory and vasoactive support, underscoring the added value of ML-based approaches over traditional scoring systems, particularly in highly unstable ICU cohorts.

The POSTCARDS study^35^ demonstrated that both ML and logistic regression models could reliably predict ICU mortality in patients with moderate-to-severe ARDS, achieving an AUROC of 0.87 in the development group and 0.91 in external validation. However, while POSTCARDS focused on a more homogenous ARDS cohort receiving lung-protective ventilation, our model addressed a broader, clinically deteriorating ICU population. Despite this increased heterogeneity, our XGBoost-based model achieved a comparable AUROC of 0.831. Additionally, while POSTCARDS identified respiratory mechanics and extrapulmonary organ dysfunction as key predictors, our model highlighted neurologic status, hematologic parameters, and age as primary prognostic factors. These findings emphasize the adaptability of ML approaches across different ICU populations and the need to tailor predictive frameworks to specific clinical contexts.

Furthermore, our results are in line with those of Ryan et al.^36^, who used XGBoost to predict mortality across ICU populations, reporting AUROCs ranging from 0.75 to 0.82 depending on cohort and prediction window. While their study focused on risk stratification over 12–72 h, our model concentrated on a fixed 24-h window post-admission. Despite these methodological differences, comparable AUROC values reinforce XGBoost’s robustness for prognostication in critically ill, high-acuity populations.

Finally, Ong et al.^37^ demonstrated that ML models, specifically XGBoost, outperformed conventional methods in predicting post-ICU outcomes among mechanically ventilated patients. Although their model targeted discharge-related endpoints, our focus was on in-ICU mortality with prediction being made at 24 h from admission. Notably, our model achieved a higher AUROC (0.831 vs. 0.693), likely reflecting differences in endpoint definition, timing, and patient acuity. Both studies, however, confirm the superior discriminatory capacity of XGBoost in high-risk ICU settings.

### Strengths and limitations

This study presents several notable strengths. It leverages AmsterdamUMCdb, a relatively underexplored European ICU dataset, providing insights from a predominantly Caucasian population and complementing U.S.-centric data such as MIMIC^38^. The use of the XGBoost algorithm, optimized with Optuna, ensures high model performance and reliability. XGBoost’s capacity to handle missing and categorical data without extensive preprocessing enhances model usability in real-world settings.

Nevertheless, several limitations should be acknowledged. In particular, the proliferation of internally validated prediction models without subsequent external validation remains a recognized challenge in the field. However, to our knowledge, this is the first ML-based model specifically targeting ICU patients requiring combined mechanical ventilation and vasoactive support, and the identification of a population-specific constellation of top predictors may generate clinically relevant hypotheses for future research.

The retrospective, observational design and lack of external validation also introduce the risk of selection bias and limit generalizability. Additionally, the requirement for 24 h of post-deterioration data excludes patients who die very early in their ICU course (n = 609, 4.9% of the initial cohort), limiting the model’s applicability to this subgroup; however, this population typically follows a fulminant clinical trajectory where prediction-based decision support offers limited added value.

Further, differences in data collection practices, variable types, missing data patterns, and outcome distributions across centers may impact model performance in external validation.

No imputation was applied, and while class imbalance was natively handled by XGBoost algorithm, both issues remain a common challenge in ICU datasets. The exclusion of unstructured data, such as clinical notes, restricted the application of natural language processing methods that could have enhanced predictive accuracy. However, this decision reduced computational requirements and facilitated model deployment on standard hardware. The absence of comorbidity data and established ICU severity scores (e.g., SAPS, APACHE) may have constrained the model’s ability to fully contextualize patient acuity and complicates direct comparisons with existing prognostic models.

We also acknowledge that treating our study cohort as a homogeneous group may overlook potential subphenotypes, and future studies could explore whether unsupervised learning can identify meaningful subgroups with distinct risk profiles.

Future work should also include temporal validation using a pre- and post-era split within the dataset to assess potential dataset shift and calibration drift, as well as evaluation of model discrimination and calibration across key subgroups (e.g., sex and age strata) to examine potential performance heterogeneity.

External validation using established ICU databases, such as MIMIC, the Salzburg Intensive Care database, or HiRID (a high–time-resolution ICU dataset), may represent the next step to assess the generalizability of the model across different patient populations and healthcare systems. Such validation would enable evaluation under varying practice patterns, case-mix distributions, and data collection processes. We acknowledge that several challenges may arise in this context, including differences in clinical practice, variable definitions, data availability and granularity. Addressing these issues will require harmonization of cohorts and features between data sources, and potentially recalibration of the model. A detailed exploration of these challenges, however, is beyond the scope of the present study and warrants dedicated investigation in the future.

We have used the default probability threshold of 0.5 for class assignment. However, since the optimal decision threshold may vary depending on the intended clinical application, the alternative cut-points could be selected in future work to prioritize sensitivity or specificity as clinically appropriate.

Another limitation concerns post-hoc calibration, where similar Brier scores suggest comparable performance, but the slight decline in calibration metrics may indicate mild overfitting.

While the model identifies high-risk patients, it does not inform treatment strategies. Therefore, emulated trials are needed, for which the model could help define eligibility criteria and identify patients most likely to benefit from targeted interventions.

Importantly, the model remains investigational and is not intended for clinical use, as it has not undergone formal safety, regulatory, or prospective validation.

### Clinical relevance

This study presents an exploratory machine learning model specifically designed for a high-risk ICU population—patients requiring both mechanical ventilation and vasopressor or inotropic support. Unlike previous models trained on broader ICU cohorts, our approach focuses on the first 24 h following the initiation of both interventions, capturing the critical early phase of physiological deterioration.

This targeted design enables early mortality prediction in a narrowly defined yet clinically urgent subgroup of ICU patients. With an AUROC of 0.831 and a macro F1 score of 0.735, the model demonstrates robust and balanced performance despite the inherent complexity of high-acuity cases. Compared to traditional tools such as the National Early Warning Score (AUROC 0.77)^34^ or ML models developed for general ICU populations^22,36^, our model demonstrates high specificity and promising discriminatory performance in the prediction of short-term ICU mortality in this high-risk subgroup.

## Conclusions

In summary, we developed an early mortality prediction model using AmsterdamUMCdb single-center data and the XGBoost algorithm, achieving robust performance in estimating in-ICU mortality after 24 h from admission in patients on both mechanical ventilation and vasoactive support. The model demonstrated in internal validation an AUROC of 0.831 (95% CI [0.799–0.860]), sensitivity of 0.492 (95% CI [0.431–0.552]), specificity of 0.934 (95% CI [0.916–0.951]), macro F1 score of 0.735 (95% CI [0.701–0.764]), and good calibration without signs of overfitting. SHAP analysis identified minimum GCS score, age, and minimum platelet count as the most influential predictors. These findings support the potential utility of interpretable ML models for high-stakes prognostication in critically ill ICU patients, although prospective validation and external replication are necessary prior to clinical implementation.

## Methods

This study was conducted and reported in accordance with the TRIPOD + AI (Transparent Reporting of a multivariable prediction model for Individual Prognosis Or Diagnosis + Artificial Intelligence) statement^39^. The completed checklist, including justifications for items marked as not applicable, is provided in Supplementary Material Table S1.

### Data source

We used the AmsterdamUMCdb, the first freely available European ICU database from the Amsterdam Medical Data Science initiative. This database comprises anonymized data of 23,106 admissions of 20,109 mixed-ICU patients hospitalized between 2003 and 2016 in the Amsterdam University Medical Center^40^.

### Study population

We utilized data from the AmsterdamUMCdb database to develop a mortality prediction model. The study population included adult ICU patients who received both mechanical ventilation and vasoactive and/or inotropic therapy either at the time of ICU admission or during the first 24 h following admission, regardless of the primary reason for admission. For the purpose of this study, the term “vasoactive” is used broadly to refer to vasopressor and/or inotropic agents^41^.

If both criteria, mechanical ventilation and vasoactive therapy, were met at ICU admission [t_0_], the model incorporated data from the entire first 24 h of the ICU stay [t_0_, (t_0_ + 24 h)]. If the criteria were met after admission, a shorter data window was used, beginning from the point at which both interventions were initiated [t_1_] and extending up to 24 h post-admission [t_1_ , (t_0_ + 24 h)].

The intended prediction time point was fixed at 24 h from ICU admission.

The model focuses on acute physiological and clinical data recorded during the early phase of critical illness, including the source ward and reason for ICU admission, laboratory parameters, ventilatory settings, vital signs, and administered medications. Due to the structure of the AmsterdamUMCdb, information on patients’ comorbidities was not available and therefore could not be included in model development^42^.

The initial query yielded 12,539 unique ICU visits from 11,715 patients, resulting in 525,415 time-series records collected at 1-min intervals.

To ensure data quality and clinical relevance, a multi-step exclusion process was applied.

First, visits ending in live discharge within 48 h of ICU admission were excluded (n = 6702), as these predominantly represented routine postoperative patients briefly admitted for post-surgical monitoring, unlikely to require sustained organ support and therefore outside the scope of this prognostic model.

Second, we excluded visits where patients died or were discharged within 24 h of the time of deterioration (n = 609), to allow sufficient time for the development of relevant clinical patterns. Third, for patients with multiple ICU admissions, only the latest episode was retained for survivors, while for deceased patients, the admission associated with death in ICU was selected (n = 412 excluded).

Overall, 61.59% of initial visits were excluded during the preprocessing stage.

After applying the above criteria, the final study cohort comprised 4816 unique ICU visits from 4816 patients, accounting for 212,449 time-series records.

**Fig. 6:**
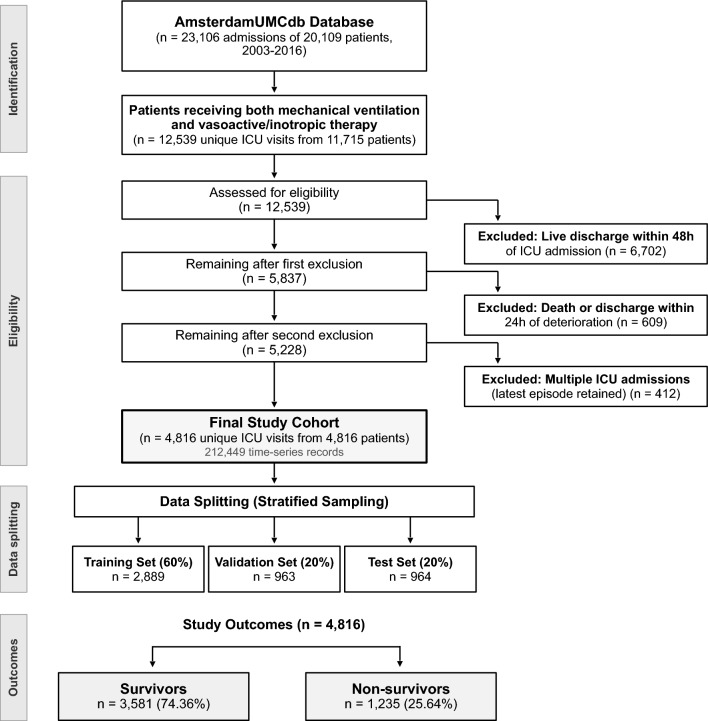
Study flow diagram. ICU, intensive care unit. Data source: AmsterdamUMCdb (Amsterdam medical data science).

The study flow diagram is presented in Fig. 6.

### Ethics

This study did not require ethical review or formal approval, as it was conducted using a fully de-identified dataset made publicly available for research purposes. Access to the AmsterdamUMCdb is granted only upon submission of an application and demonstration of appropriate qualifications, including documented expertise in intensive care medicine and proof of completion of mandatory training (e.g., Data or Specimens Only Research or equivalent).

The dataset adheres to all relevant European and Dutch data protection regulations, including the General Data Protection Regulation (GDPR). A Data Privacy Impact Assessment was performed, and an independent evaluation led by experts in medical ethics and data privacy concluded that the risk of re-identification is minimal. As such, the dataset is considered anonymous under the GDPR.

Further details regarding the legal, ethical, and privacy framework governing the dataset are available at: https://amsterdammedicaldatascience.nl/.

All methods were performed in accordance with the relevant guidelines and regulations.

### Data pre-processing

Outliers were identified using interquartile range (IQR) method, and handled with mean-replacement.

Time-series data of physiological variables: heart rate (HR), systolic blood pressure (SBP), diastolic blood pressure (DBP) and mean arterial pressure (MAP), measured for each patient over the initial 24 h following admission to the ICU, were aggregated into one prediction time-point and a single value, with the Time-Weighted Average (TWA) method. For each of these variables TWA was calculated based on the depth and time exceeding a predefined upper- or lower-bound threshold.

The weighted sum of the distance of the each value from the threshold, scaled by the time difference between consecutive measurements, and normalized by the total observation time, was calculated according to the formula:

$$TWA = \frac{{\mathop \sum \nolimits_{i = 1}^{n} \left( {f\left( {xi,T} \right) \times \left( {t_{{\mathrm{i}}} - t_{{{\mathrm{i}} - 1}} } \right)} \right)}}{{\mathop \sum \nolimits_{i = 1}^{n} \left( {t_{{\mathrm{i}}} - t_{{{\mathrm{i}} - 1}} } \right)}}$$

where:

x_i_ = the value of the variable at time t_i_.

T = the upper/lower bound threshold.

$f\left( {x_{i} ,T} \right)$ = a function defining distance from the threshold; $\max \left( {0,x_{i} - T} \right)$ for values above the threshold, and $\max \left( {0,T - x_{i} } \right)$ for values below the threshold.

t_i_ = the time of the i-th measurement.

t_i−1_ = the time of the (i-th—1, i.e. previous) measurement.

n = number of all measurements.

$\max \left( {x_{i} - T} \right)$ = the “depth” above the threshold; the expression takes “0” value if the value x_i_ is below the threshold.

The reference thresholds were defined as follows: upper-bound for HR = 100 bpm, lower-bound = 90, 60 and 65 mmHg for SBP, DBP and MAP, respectively.

For the remaining numerical data, including laboratory results, medical scales, and less-frequent physiological measurements, aggregation was guided by domain knowledge. Depending on the clinical relevance of each variable, the minimum, maximum, or mean value recorded over a 24-h period post-admission was used to create a representative summary.

The operational definitions of aggregated predictors are presented in Supplementary Material Table S2.

To handle missing data, columns with more than 70% missing values were identified and removed from the aggregated dataset. This resulted in the exclusion of 10 variables: ‘SVR_min’, ‘SAPS_max’, ‘SVV_max’, ‘PCT_max’, ‘Fibrinogen_min’, ‘PCWP_max’,’CO_min’, ‘NEUTROPH_max’, ‘Mean_PAP_max’ and ‘NT_proBNP_max’. The threshold was chosen to minimize the impact of sparsity on model performance while retaining the most informative features. This step generated the fully aggregated dataset with overall 22.7% missing data. We opted not to use imputation for missing data for two primary reasons: to preserve dataset integrity and to prevent potential bias that could arise if certain patient groups lacked recorded data due to the absence of a measurement indication during the study period. Additionally, XGBoost natively handles missing data. The missingness report is presented in Supplementary Material Table S3.

### Data splitting

The dataset was split into training (60%, n = 2889), validation (20%, n = 963), and test (20%, n = 964) sets. Data splitting was performed using stratified sampling based on the outcome variable to preserve class distribution across all sets.

The class distribution was consistent across training, validation, and test sets, with negative-to-positive sample ratios of 2.899, 2.899, and 2.903, respectively.

As only one ICU admission per patient was retained, data splitting was performed at the patient level, ensuring independence between the training, validation, and test sets.

### Predictors

For model development, 66 features were utilized, all accessible within 24 h from admission to the ICU (in patients on mechanical ventilation and vasoactive therapy), encompassing time-series data ‘on vital signs, laboratory parameters, medical scales, administered drugs, and admission reasons. The baseline characteristics of predictors used for training of the model is presented in Table 1.

### Outcome

The model’s outcome was in-ICU mortality, a binary variable where 0 represents survival to ICU discharge and 1 indicates death within the ICU. The class imbalance ratio was 2.9, with the majority of patients in the class 0 (3581 survivors vs 1235 non-survivors). To quantify differences between survivors and non-survivors standardized mean differences (SMD) are reported; values ≥ 0.1 indicate meaningful group differences.

### Machine learning framework and development

We used an XGBoost machine-learning algorithm^43^ to develop our model.

A fixed random seed was applied for data splitting, cross-validation, and model initialization to ensure reproducibility.

The baseline model training was performed on 2889 patients from the training set, using all 66 predictors. Stratified tenfold cross-validation, repeated 5 times, was used during model training, which allowed for robust model assessment, with preservation of class distribution within the folds.

In the next step, the model’s hyperparameters were fine-tuned using the validation set. We used the Optuna optimization framework^44^ to efficiently search the hyperparameter space and identify their combinations that maximize the objective, the area under receiver operating characteristic (AUROC). The Optuna search process was configured for 1000 trials; the hyperparameters, their ranges and the tuning results are presented in Supplementary Material Figure S1.

Subsequently, the final model was trained with Optuna hyperparameters, using the training set and cross-validation as described for the baseline model training.

Finally, we used SHapley Additive exPlanations (SHAP). SHAP is a powerful technique which allows for gaining insight into the model’s prediction process^45^. Whereas XGBoost offers increased flexibility and often outperforms other available algorithms for tasks involving tabular data, it is commonly regarded as a “black-box” model, where greater efficiency comes at the cost of reduced interpretability. Thus, utilizing SHAP technique aligns with the explainable AI paradigm, and may enhance stakeholders’ trust in machine learning-based solutions^46^.

### Model evaluation

The model’s discriminatory performance was evaluated based on a test set with data from 964 patients. To assess model’s capacity to differentiate between classes, we used the following metrics: area under receiver operating characteristic (AUROC), sensitivity (true positive rate (TPR)), specificity (true negative rate (TNR)), positive predictive value (PPV), negative predictive value (NPV) and macro F1 score. We obtained averages for the performance metrics and estimated confidence intervals (CI) using the bootstrapping procedure with 1000 replications on the test set.

Calibration of probabilistic predictions was assessed using calibration curves and quantified by the Brier score (range 0–1, where 0 indicates perfect agreement between predicted probabilities and observed outcomes and 1 complete discrepancy). To evaluate whether post-hoc recalibration improved probability estimates, Platt scaling (sigmoid) and isotonic regression were applied using five-fold cross-validation on the training set with a fixed random seed to ensure reproducibility. Calibration was further quantified using calibration-in-the-large (intercept) and calibration slope, obtained by fitting a logistic regression model to the logit-transformed predicted probabilities. An intercept of 0 and slope of 1 indicate ideal calibration, with deviations reflecting systematic bias and over- or underfitting, respectively. These measures were calculated for the uncalibrated as well as the recalibrated models. For all metrics, including Brier score, calibration slope, and intercept, 95% CI were estimated using 1000 bootstrap replications on the independent test set.

The SHapley Additive exPlanations (SHAP) analysis was used together with the final model and test data to illustrate the model’s decision-making.

The model’s ability to generalize to new data (internal validation) was illustrated with the learning curves, which depict the training and validation errors, thus showing the risk of over/underfitting (bias-variance relationship).

### Programming environment

We used Google Colab 0.0.1a2, Python 3.11.11, scikit-learn 1.6.1, XGBoost 2.1.4, Optuna 4.2.1 and SHAP 0.47.1.

## Supplementary Information

Supplementary Information.

## Data Availability

The dataset analyzed in this study is available from https://amsterdammedicaldatascience.nl/#amsterdamumcdb but requires application, credentialing, and acceptance of a data use agreement.

## Code availability

The code used for model development and analysis is available from the corresponding author upon reasonable request. Access may be subject to institutional and data protection considerations.

## Declarations

### Declaration of use of AI-based tools

During the writing process, the author(s) used GPT-5 in order to improve language and readability. After using this tool, the author(s) reviewed and manually edited the content as required and take(s) full responsibility for the content of the publication.
