# Supplementary material for: Early prognostication for ICU patients with combined respiratory and circulatory failure: an interpretable machine learning approach
Source: Sci Rep. 2026 Jun 25;16:21423. doi: 10.1038/s41598-026-59064-y (PMC13351017; doi:10.1038/s41598-026-59064-y)

Table S1.TRIPOD+AI checklist for the reporting of prediction model studies.

Section	Item	D/E	Checklist Item	Reported on Page
**TITLE**	1	D;E	Identify the study as developing or evaluating the performance of a multivariable prediction model, the target population, and the outcome to be predicted	Page 1
**ABSTRACT**	2	D;E	See TRIPOD+AI for Abstracts checklist	Page 1
**BACKGROUND**	3a	D;E	Explain the healthcare context (including whether diagnostic or prognostic) and rationale for developing or evaluating the prediction model, including references to existing models	Pages 1-2
	3b	D;E	Describe the target population and the intended purpose of the prediction model in the context of the care pathway, including its intended users (eg, healthcare professionals, patients, public)	Pages 2, 7
	3c	D;E	Describe any known health inequalities between sociodemographic groups	**N/A**
**OBJECTIVES**	4	D;E	Specify the study objectives, including whether the study describes the development or validation of a prediction model (or both)	Page 2
**DATA**	5a	D;E	Describe the sources of data separately for the development and evaluation datasets (eg, randomised trial, cohort, routine care or registry data), the rationale for using these data, and representativeness of the data	Pages 7-8
	5b	D;E	Specify the dates of the collected participant data, including start and end of participant accrual; and, if applicable, end of follow-up	Page 7
**PARTICIPANTS**	6a	D;E	Specify key elements of the study setting (eg, primary care, secondary care, general population) including the number and location of centres	Pages 7-8
	6b	D;E	Describe the eligibility criteria for study participants	Pages 7-8
	6c	D;E	Give details of any treatments received, and how they were handled during model development or evaluation, if relevant	Pages 7-8
**DATA PREPARATION**	7	D;E	Describe any data pre-processing and quality checking, including whether this was similar across relevant sociodemographic groups	Pages 8-9
**OUTCOME**	8a	D;E	Clearly define the outcome that is being predicted and the time horizon, including how and when assessed, the rationale for choosing this outcome, and whether the method of outcome assessment is consistent across sociodemographic groups	Page 10
	8b	D;E	If outcome assessment requires subjective interpretation, describe the qualifications and demographic characteristics of the outcome assessors	**N/A**
	8c	D;E	Report any actions to blind assessment of the outcome to be predicted	**N/A**
**PREDICTORS**	9a	D	Describe the choice of initial predictors (eg, literature, previous models, all available predictors) and any pre-selection of predictors before model building	Page 9
	9b	D;E	Clearly define all predictors, including how and when they were measured (and any actions to blind assessment of predictors for the outcome and other predictors)	Pages 9, 15-16
	9c	D;E	If predictor measurement requires subjective interpretation, describe the qualifications and demographic characteristics of the predictor assessors	**N/A**
**SAMPLE SIZE**	10	D;E	Explain how the study size was arrived at (separately for development and evaluation), and justify that the study size was sufficient to answer the research question. Include details of any sample size calculation	**N/A**
**MISSING DATA**	11	D;E	Describe how missing data were handled. Provide reasons for omitting any data	Page 9
**ANALYTICAL METHODS**	12a	D	Describe how the data were used (eg, for development and evaluation of model performance) in the analysis, including whether the data were partitioned, considering any sample size requirements	Page 9

	12b	D	Depending on the type of model, describe how predictors were handled in the analyses (functional form, rescaling, transformation, or any standardisation)	Pages 8-9
	12c	D	Specify the type of model, rationale, all model building steps, including any hyperparameter tuning, and method for internal validation	Pages 10-11
	12d	D;E	Describe if and how any heterogeneity in estimates of model parameter values and model performance was handled and quantified across clusters (eg, hospitals, countries). See TRIPOD-Cluster for additional considerations	**N/A**
	12e	D;E	Specify all measures and plots used (and their rationale) to evaluate model performance (eg, discrimination, calibration, clinical utility) and, if relevant, to compare multiple models	Pages 10-11
	12f	E	Describe any model updating (eg, recalibration) arising from the model evaluation, either overall or for particular sociodemographic groups or settings	Pages 10-11
	12g	E	For model evaluation, describe how the model predictions were calculated (eg, formula, code, object, application programming interface)	**N/A**
**CLASS IMBALANCE**	13	D;E	If class imbalance methods were used, state why and how this was done, and any subsequent methods to recalibrate the model or the model predictions	Page 6
**FAIRNESS**	14	D;E	Describe any approaches that were used to address model fairness and their rationale	**N/A**
**MODEL OUTPUT**	15	D	Specify the output of the prediction model (eg, probabilities, classification). Provide details and rationale for any classification and how the thresholds were identified	Pages 3, 6, 10
**TRAINING VERSUS EVALUATION**	16	D;E	Identify any differences between the development and evaluation data in healthcare setting, eligibility criteria, outcome, and predictors	**N/A**
**ETHICAL APPROVAL**	17	D;E	Name the institutional research board or ethics committee that approved the study and describe the participant informed consent or the ethics committee waiver of informed consent	Page 8
**FUNDING**	18a	D;E	Give the source of funding and the role of the funders for the present study	Page 14
**CONFLICTS OF INTEREST**	18b	D;E	Declare any conflicts of interest and financial disclosures for all authors	Page 14
**PROTOCOL**	18c	D;E	Indicate where the study protocol can be accessed or state that a protocol was not prepared	**N/A**
**REGISTRATION**	18d	D;E	Provide registration information for the study, including register name and registration number, or state that the study was not registered	**N/A**
**DATA SHARING**	18e	D;E	Provide details of the availability of the study data	Page 11
**CODE SHARING**	18f	D;E	Provide details of the availability of the analytical code	Page 11
**PATIENT AND PUBLIC INVOLVEMENT**	19	D;E	Provide details of any patient and public involvement during the design, conduct, reporting, interpretation, or dissemination of the study or state no involvement	**N/A**
**RESULTS - PARTICIPANTS**	20a	D;E	Describe the flow of participants through the study, including the number of participants with and without the outcome and, if applicable, a summary of the follow-up time. A diagram may be helpful	Pages 3, 8
	20b	D;E	Report the characteristics overall and, where applicable, for each data source or setting, including the key dates, key predictors (including demographics), treatments received, sample size, number of outcome events, follow-up time, and amount of missing data. A table may be helpful. Report any differences across key demographic groups	Pages 2-3, 15-16
	20c	E	For model evaluation, show a comparison with the development data of the distribution of important predictors (demographics, predictors, and outcome)	**N/A**
**RESULTS - MODEL DEVELOPMENT**	21	D;E	Specify the number of participants and outcome events in each analysis (eg, for model development, hyperparameter tuning, model evaluation)	Pages 4, 9

**RESULTS - MODEL SPECIFICATION**	22	D	Provide details of the full prediction model (eg, formula, code, object, application programming interface) to allow predictions in new individuals and to enable third party evaluation and implementation, including any restrictions to access or reuse (eg, freely available, proprietary)	Page 11
**RESULTS - MODEL PERFORMANCE**	23a	D;E	Report model performance estimates with confidence intervals, including for any key subgroups (eg, sociodemographic). Consider plots to aid presentation	Pages 3-4
	23b	D;E	If examined, report results of any heterogeneity in model performance across clusters. See TRIPOD-Cluster for additional details	**N/A**
**RESULTS - MODEL UPDATING**	24	E	Report the results from any model updating, including the updated model and subsequent performance	Pages 3-4
**DISCUSSION - INTERPRETATION**	25	D;E	Give an overall interpretation of the main results, including issues of fairness in the context of the objectives and previous studies	Pages 4-7
**DISCUSSION - LIMITATIONS**	26	D;E	Discuss any limitations of the study (such as a non-representative sample, sample size, overfitting, missing data) and their effects on any biases, statistical uncertainty, and generalisability	Page 6
**DISCUSSION - USABILITY**	27a	D	Describe how poor quality or unavailable input data (eg, predictor values) should be assessed and handled when implementing the prediction model	**N/A**
	27b	D	Specify whether users will be required to interact in the handling of the input data or use of the model, and what level of expertise is required of users	**N/A**
	27c	D;E	Discuss any next steps for future research, with a specific view to applicability and generalisability of the model	Pages 6-7

Legend: D = Development only | E = Evaluation only | D;E = Both | **N/A** = Not reported in manuscript
Justification for items marked as N/A (in accordance with TRIPOD+AI guidelines):

- Items 3c, 14 (Health inequalities & Fairness): Due to the retrospective, single-center design and the structure of the AmsterdamUMCdb, information on race, ethnicity, and socioeconomic status was not available. Consequently, a formal assessment of health inequalities between sociodemographic groups or a dedicated fairness analysis of model performance across such groups could not be performed.
- Item 10 (Sample size): No formal sample size calculation was performed prior to model development. The study used all eligible patients from the AmsterdamUMCdb who met the inclusion criteria, resulting in a final cohort of 4,816 patients with 1,235 outcome events. A post hoc sample size justification was not pursued; however, the number of events per variable (EPV) ratio was approximately 18.7 (1,235 events / 66 predictors), exceeding commonly recommended minimum thresholds for prediction model development.
- Items 8b, 8c, 9c (Subjective assessments & Blinding): Not applicable. The outcome (ICU mortality) and all predictors consist of objective data automatically extracted from electronic health records ; subjective interpretation and blinding were not relevant to this study design.
- Items 12d, 23b (Clusters): The study is based on a single-center database. Evaluating heterogeneity or model performance across clusters (e.g., multiple hospitals) was not applicable.
- Items 16, 20c (Training vs Evaluation data differences): As this is a single-center development study with internal validation only, no formal comparison between development and external evaluation datasets was applicable.
- Items 18c, 18d, 19 (Protocol, Registration, & PPI): No study protocol was prepared or registered prior to the conduct of this study. Patient and public involvement was not incorporated into the design, conduct, or dissemination of this retrospective database study.
- Items 27a, 27b (Model implementation & Usability): Handling of poor-quality or unavailable input data at the time of potential model implementation, as well as the level of user expertise required for model use, were not formally assessed. The model remains investigational and is not intended for clinical deployment in its current form.

Table S2. Operational definitions of aggregated predictors.

Predictor	Operational definition
HR_twa_over	Time-weighted average of heart rate above 100 bpm within the observation window*; unit: bpm
SBP_twa_under	Time-weighted average of systolic blood pressure below 90 mmHg within the observation window; unit: mmHg
DBP_twa_under	Time-weighted average of diastolic blood pressure below 60 mmHg within the observation window; unit: mmHg
MAP_twa_under	Time-weighted average of mean arterial pressure below 65 mmHg within the observation window; unit: mmHg
SpO2_min	Minimum peripheral oxygen saturation (%) recorded within the observation window
Temperature_max	Maximum body temperature (°C) within the observation window
FiO2_max	Maximum fraction of inspired oxygen (%) within the observation window
PEEP_max	Maximum positive end-expiratory pressure (cmH_2O) within the observation window
PPeak_max	Maximum peak airway pressure (cmH_2O) within the observation window
MV_mean	Mean minute ventilation (L/min) within the observation window
PaO2_FiO2_ratio_min	Minimum ratio of arterial oxygen partial pressure to inspired oxygen fraction (mmHg) within the observation window
CVP_min	Minimum central venous pressure (mmHg) within the observation window

Urine_output_min	Minimum hourly urine output (ml/h) within the observation window
GCS_min	Minimum Glasgow Coma Scale score within the observation window
RASS_scale_min	Minimum Richmond Agitation–Sedation Scale score within the observation window
RASS_scale_max	Maximum Richmond Agitation–Sedation Scale score within the observation window
Ramsay_scale_min	Minimum Ramsay Sedation Scale score within the observation window
Ramsay_scale_max	Maximum Ramsay Sedation Scale score within the observation window
pH_min	Minimum arterial pH within the observation window
pO2_min	Minimum arterial oxygen partial pressure (mmHg) within the observation window
pCO2_max	Maximum arterial carbon dioxide partial pressure (mmHg) within the observation window
HCO3_min	Minimum bicarbonate concentration (mmol/L) within the observation window
Lactate_max	Maximum serum lactate (mmol/L) within the observation window
Glucose_mean	Mean blood glucose concentration (mmol/L) within the observation window
Sodium_mean	Mean serum sodium concentration (mmol/L) within the observation window

Potassium_mean	Mean serum potassium concentration (mmol/L) within the observation window
Phosphate_min	Minimum serum phosphate concentration (mmol/L) within the observation window
Chloride_min	Minimum serum chloride concentration (mmol/L) within the observation window
Creatinine_max	Maximum serum creatinine (µmol/L) within the observation window
Hemoglobin_min	Minimum hemoglobin concentration (mmol/L) within the observation window
LEU_max	Maximum leukocyte count ($\times 10^9$/L) within the observation window
LYMPH_max	Maximum lymphocyte count ($\times 10^9$/L) within the observation window
MONOCYT_max	Maximum monocyte count ($\times 10^9$/L) within the observation window
PLT_min	Minimum platelet count ($\times 10^9$/L) within the observation window
INR_max	Maximum international normalized ratio within the observation window
APTT_max	Maximum activated partial thromboplastin time (seconds) within the observation window
Bilirubin_total_max	Maximum total bilirubin concentration (µmol/L) within the observation window
ALAT_max	Maximum alanine aminotransferase (U/L) within the observation window

ASPAT_max	Maximum aspartate aminotransferase (U/L) within the observation window
Albumin_min	Minimum serum albumin concentration (g/L) within the observation window
Troponin_max	Maximum cardiac troponin concentration (µg/L) within the observation window
Creatine_kinase_max	Maximum creatine kinase level (U/L) within the observation window
CRP_max	Maximum C-reactive protein concentration (mg/L) within the observation window

*Observation window: predictors were aggregated within a patient-specific observation window during the first 24 hours of ICU stay. If both deterioration criteria, mechanical ventilation and vasoactive therapy, were present at ICU admission [t_0], predictor values were aggregated over the full 24-hour period [t_0–t_0+24 h]. If the criteria were met after admission [t_1], aggregation began at the time both interventions were first present and continued until 24 hours after ICU admission [t_1–(t_0+24 h)]. Continuous physiologic variables were summarized using clinically informed aggregation rules (minimum, maximum, or mean) over the observation window. Hemodynamic instability was quantified using time-weighted exposure relative to predefined thresholds (TWA - time-weighted average).

Table S3. Missingness report across all the candidate features.

Column	NaN_Percentage
SVR_min	100.00
SAPS_max	99.98
SVV_max	99.15
PCT_max	97.97
Fibrinogen_min	93.11
PCWP_max	80.25
CO_min	80.00
NEUTROPH_max	78.65
Mean_PAP_max	76.29
NT_proBNP_max	73.84
MONOCYT_max	66.05
LYMPH_max	66.01
PaO2_FiO2_ratio_min	58.22
RASS_scale_min	56.94
RASS_scale_max	56.94
Troponin_max	52.97
Admission_reason	52.93
Ceftriaxone	51.79
Colistin	51.79
Fluconazole	51.79
Linezolid	51.79
Ciprofloxacin	51.79
Amikacin	51.79
Meropenem	51.79
Imipenem	51.79
Vancomycin	51.79
Anidulafungin	51.79
Metronidazole	51.79

Gentamicin	51.79
Voriconazole	51.79
Hydrocortisone	51.79
Albumin_iv	51.79
Creatine_kinase_max	39.58
Chloride_min	35.94
CRP_max	31.83
CVP_min	24.07
Bilirubin_total_max	22.86
Ramsay_scale_max	22.26
Ramsay_scale_min	22.26
Temperature_max	19.52
ASPAT_max	17.05
ALAT_max	16.32
Albumin_min	14.60
Lactate_max	12.94
GCS_min	10.74
MV_mean	8.12
PPeak_max	5.81
PEEP_max	5.61
FiO2_max	5.40
INR_max	4.53
Phosphate_min	3.32
Urine_output_min	2.26
APTT_max	2.22
LEU_max	1.27
Creatinine_max	1.18
PLT_min	1.14
Transfusion	0.60
Glucose_mean	0.46
pO2_min	0.31
Sodium_mean	0.29

HCO3_min	0.29
Hemoglobin_min	0.29
Potassium_mean	0.29
pH_min	0.27
Ventilation_mode_category	0.27
pCO2_max	0.27
SBP_twa_under	0.06
HR_twa_over	0.06
DBP_twa_under	0.06
SpO2_min	0.06
MAP_twa_under	0.06
Specialty_category	0.00
Age	0.00
Gender	0.00
Noradrenaline_mg_per_hour_max	0.00
Time_from_admission_to_deterioration_mean	0.00
Died	0.00

For model building only variables with <70% of missing values have been included. Excluded variables were: 'SVR_min', 'SAPS_max', 'SVV_max', 'PCT_max', 'Fibrinogen_min', 'PCWP_max', 'CO_min', 'NEUTROPH_max', 'Mean_PAP_max' and 'NT_proBNP_max'.

Figure S1. Hyperparameter tuning report

a)

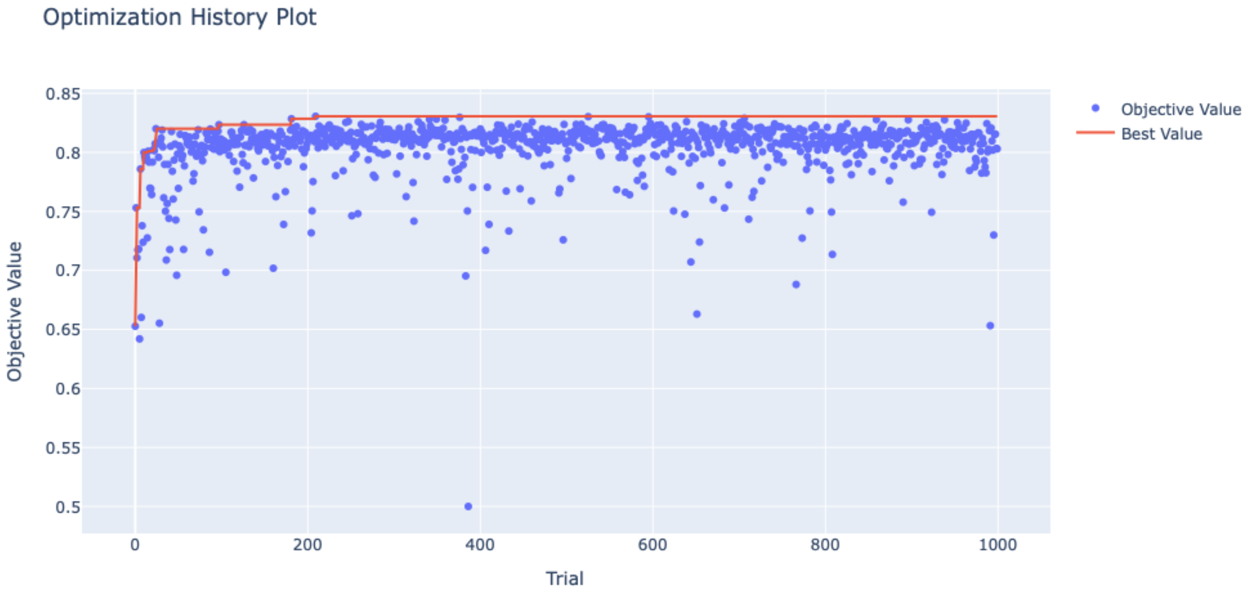

b)

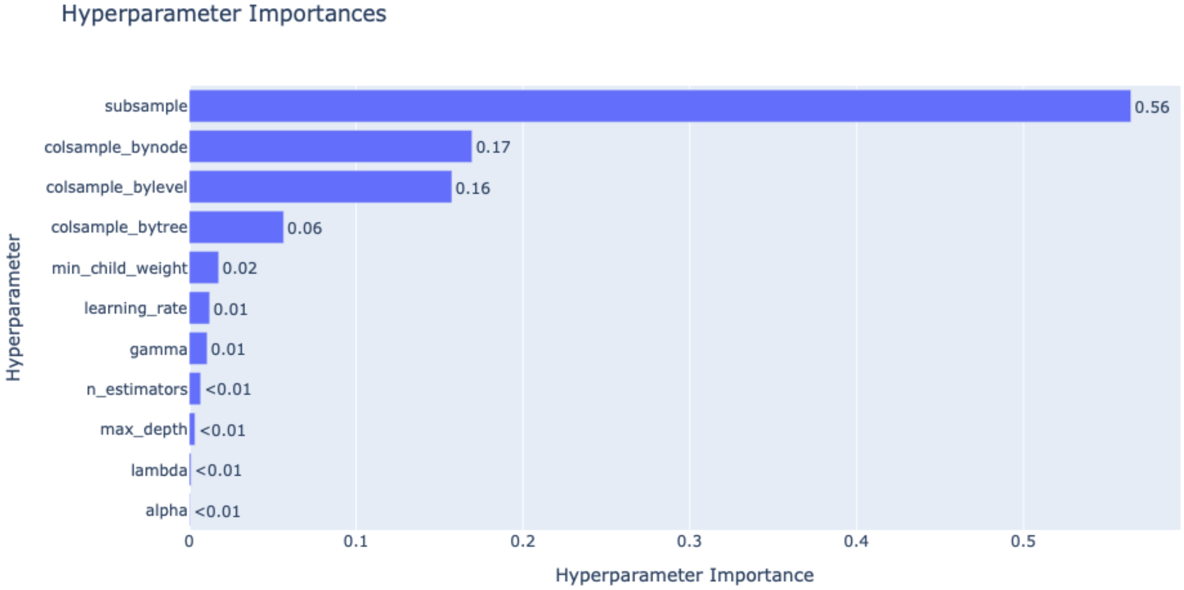

c)

Hyperparameter	Value	Search Space
max_depth	6.0	int [2, 10]
learning_rate	0.210018086932273	float [1e-3, 0.3] (log)
subsample	0.983011191939844	float [0.05, 1]
colsample_bytree	0.886299784464055	float [0.05, 1]
colsample_bylevel	0.814043309294259	float [0.05, 1]
colsample_bynode	0.977568143850154	float [0.05, 1]
min_child_weight	19	int [1, 20]
lambda	2.69126358293887×10^-05	float [1e-8, 1.0] (log)
alpha	0.106510620726277	float [1e-8, 1.0] (log)
gamma	1.34008505017868	float [0, 5]
n_estimators	213	int [5, 1000]

a) optimization history, b) hyperparameter importances and c) hyperparameter range space with their optimal values from Optuna obtained from running the study with 1000 trials.

Figure S2. Balance-variance trade-off

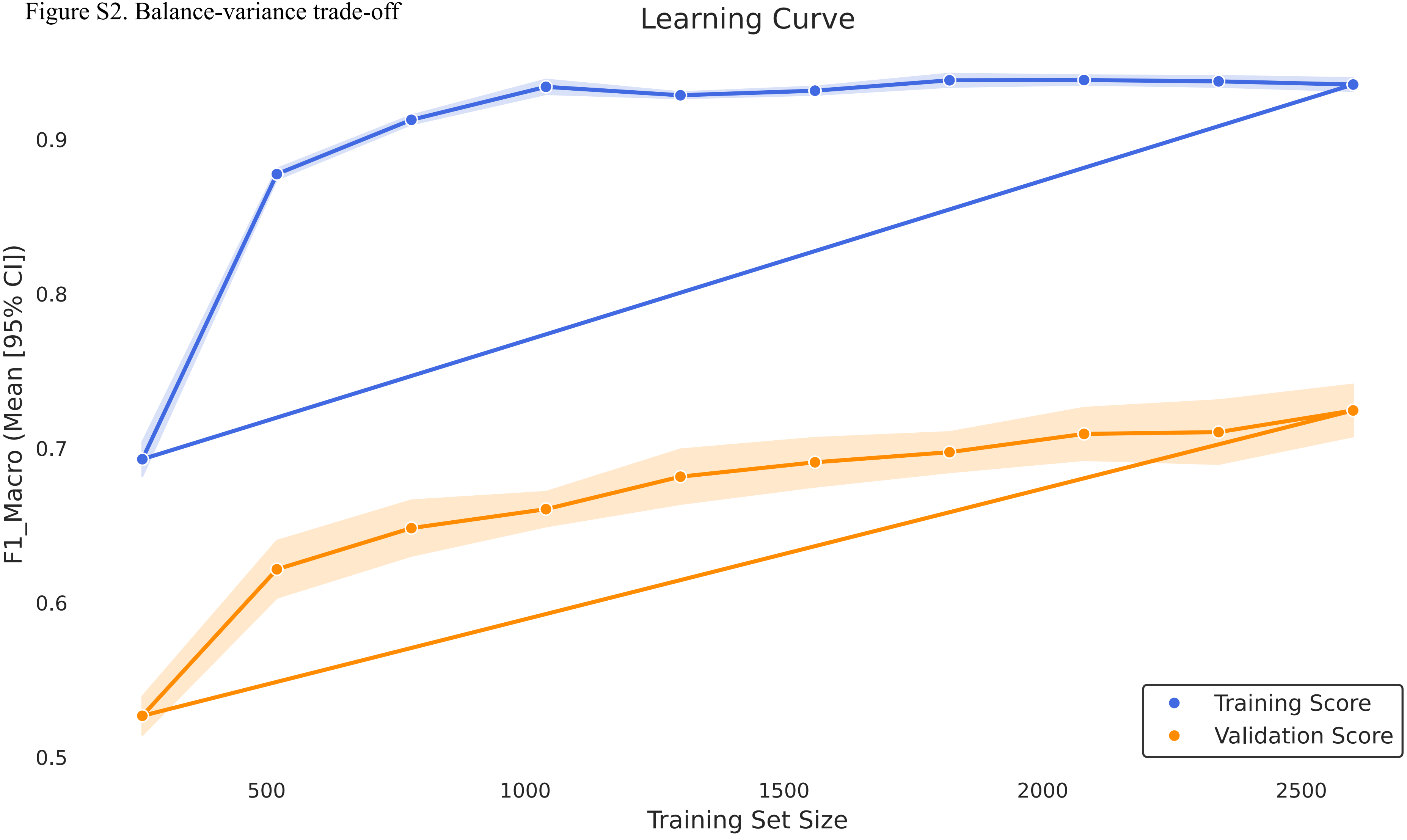

Supplement: Supplementary file 1 — Supplementary Information. [file 41598_2026_59064_MOESM1_ESM.pdf]
